# Prepectoral Versus Subpectoral Implant-Based Breast Reconstruction: A Systemic Review and Meta-analysis

**DOI:** 10.1245/s10434-022-12567-0

**Published:** 2022-10-16

**Authors:** Edvin Ostapenko, Larissa Nixdorf, Yelena Devyatko, Ruth Exner, Kerstin Wimmer, Florian Fitzal

**Affiliations:** 1 Department of General Surgery, Comprehensive Cancer Center Vienna, Medical University of Vienna, Vienna, Austria; 2 Faculty of Medicine, Vilnius University, Vilnius, Lithuania

## Abstract

**Background:**

Implant-based breast reconstruction (IBBR) remains the standard and most popular option for women undergoing breast reconstruction after mastectomy worldwide. Recently, prepectoral IBBR has resurged in popularity, despite limited data comparing prepectoral with subpectoral IBBR.

**Methods:**

A systematic search of PubMed and Cochrane Library from January 1, 2011 to December 31, 2021, was performed following the Preferred Reporting Items for Systematic reviews and Meta-Analyses (PRISMA) reporting guidelines, data were extracted by independent reviewers. Studies that compared prepectoral with subpectoral IBBR for breast cancer were included.

**Results:**

Overall, 15 studies with 3,101 patients were included in this meta-analysis. Our results showed that patients receiving prepectoral IBBR experienced fewer capsular contractures (odds ratio [OR], 0.54; 95% confidence interval [CI], 0.32–0.92; *P* = 0.02), animation deformity (OR, 0.02; 95% CI, 0.00–0.25; *P* = 0.002), and prosthesis failure (OR, 0.58; 95% CI, 0.42–0.80; *P* = 0.001). There was no significant difference between prepectoral and subpectoral IBBR in overall complications (OR, 0.83; 95% CI, 0.64–1.09; *P* = 0.19), seroma (OR, 1.21; 95% CI, 0.59-2.51; *P* = 0.60), hematoma (OR, 0.76; 95% CI, 0.49–1.18; *P* = 0.22), infection (OR, 0.87; 95% CI, 0.63–1.20; *P* = 0.39), skin flap necrosis (OR, 0.70; 95% CI, 0.45–1.08; *P* = 0.11), and recurrence (OR, 1.31; 95% CI, 0.52–3.39; *P* = 0.55). Similarly, no significant difference was found in Breast-Q scores between the prepectoral and subpectoral IBBR groups.

**Conclusions:**

The results of our systematic review and meta-analysis demonstrated that prepectoral, implant-based, breast reconstruction is a safe modality and has similar outcomes with significantly lower rates of capsular contracture, prosthesis failure, and animation deformity compared with subpectoral, implant-based, breast reconstruction.

**Supplementary Information:**

The online version contains supplementary material available at 10.1245/s10434-022-12567-0.

In 2020, there were 2.3 million women diagnosed with breast cancer and 685,000 deaths globally. As of the end of 2020, there were 7.8 million women alive who were diagnosed with breast cancer in the past 5 years, making it the world’s most prevalent cancer.^1,2^ The rate of women who undergo breast reconstruction after mastectomy every year increases due to better aesthetic outcomes and quality of life (QoL). Implant-based breast reconstruction (IBBR) remains the most common reconstructive approach.^3,4^ The selection of the implant plane during breast reconstruction has recently become a subject of debate. Prepectoral IBBR involves filling the space between the pectoralis major muscle and mastectomy skin flap, whereas subpectoral position involves placing the implant between the pectoralis major muscle and chest wall. First described in the 1970s, the prepectoral IBBR technique was associated with unacceptably high rate of complications, including infection, implant exposure, capsular contracture.^5^ To decrease the risk of complication, the procedure has been modified to position the implant subpectorally. The subpectoral IBBR was a reliable and safe alternative. In the past few years, prepectoral IBBR has resurged in popularity. Modern iterations have demonstrated improved outcomes for several reasons, including a better clinical understanding of mastectomy flap perfusion, new reconstructive techniques as well as the introduction of new generation implants, which are linked to decreased capsular contracture and have allowed safe and efficacious prepectoral implant placement.^6–8^ The benefits, risks, and clinical outcomes between prepectoral and subpectoral IBBR are now actively investigated in prospective randomized, clinical trials, such as the PREPEC OPBC-02.

The purpose of this systematic review and meta-analysis was to assess and compare the clinical outcomes and efficacy between prepectoral and subpectoral implant-based breast reconstruction.

## Methods

This systematic review and meta-analysis was conducted in accordance with the Preferred Reporting Items for Systematic Reviews and Meta-analyses (PRISMA) standards,^9^ and the a priori protocol was registered in the PROSPERO database (CRD42022312094).

### Literature Search and Search Criteria

The systematic review was conducted using PubMed and the Cochrane Library for studies published between January 1, 2011 and December 31, 2021 (eMethods in the *Supplement*). The inclusion criteria were as follows: (1) reporting follow-up for at least 1 year; (2) the article described implant-based breast reconstructions with implant places either prepectorally or subpectorally; (3) publication was from January 1, 2011 to December 31, 2021; (4) the full text was available; (5) reporting of relevant outcomes, i.e., postoperative complications; and (6) studies published in English.

The exclusion criteria were as follows: (1) studies evaluating <60 patients; (2) abstracts; (3) patients undergoing other breast reconstruction operations; and (4) insufficient data or not meeting our inclusion criteria.

### Data Extraction

Data for the analysis of prepectoral implant-based breast reconstruction (IBBR) versus subpectoral implant-based breast reconstruction (IBBR) were extracted independently by two reviewers (E.O. and F.F.); disagreements were resolved through discussion. The data extracted from each study, including year of publication, country of origin, patient demographics, such as gender, mean age, follow-up time, operative details, type of breast reconstruction, and main outcomes, were collated using a standardized form. Attempts were made to contact the corresponding author to clarify missing data in any of the included studies. Data were inputted into RevMan 5.4 software for analysis.^10^

### Risk-of-Bias and Publication Bias Assessment

We assessed for risk of bias using the Cochrane Risk of Bias In Nonrandomized studies of Interventions (ROBINS-I) tool.^11^ The assessment was recorded as low, moderate, serious, critical risk of bias, or no information. The degree of bias was measured using the Egger bias test.

### Statistical Analysis

Data were analyzed using the Review Manager Version 5.4 (The Nordic Cochrane Center, The Cochrane Collaboration, Copenhagen, Denmark) and STATA Version 16.0 (Stata Corporation, College Station, TX).^10,12^ Odds ratios (OR) and its associated 95% confidence interval (CI) were measured. Statistical heterogeneity was tested by using Chi-square and inconsistency (*I*^2^) statistics. *I*^2^ value ranging from 0 to 100% were used to quantify the effect of heterogeneity. *I*^2^ value ≥ 40% represented significant heterogeneity and pooled odds ratios (OR) were estimated using a random-effect model (DerSimonian and Laird method).^13^ When no statistical heterogeneity was observed (*I*^2^ value < 40%), a fixed effects model (Mantel-Haenszel method) was used.^14^ Publication bias was evaluated using Egger regression tests. *P* value < 0.05 was considered a statistically significant difference between the two groups.

## Results

### Study Screening

**Fig. 1:**
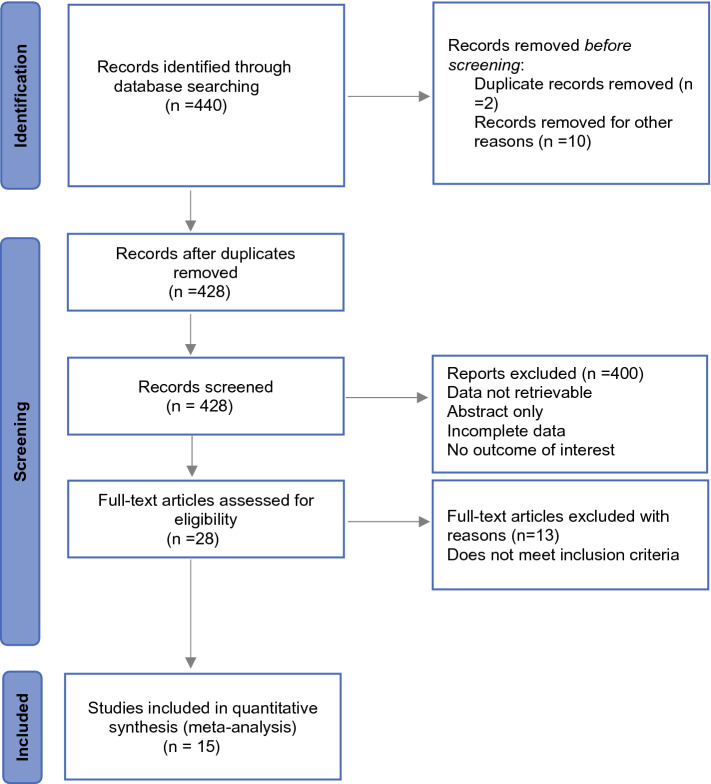
Flow diagram of literature search and selection of included studies for meta-analysis

The study flow diagram is depicted in (Fig. 1). In total, 440 studies were initially identified; after duplicates were removed, the titles and abstracts of 428 studies were screened. Of these, 400 studies were excluded, and the full texts of the remaining 28 studies were obtained for further evaluation. After reading the full texts, 13 studies were excluded for various reasons, including incorrect comparisons, short follow-up time, and inappropriate numerical data necessary for statistical analysis. Ultimately, 15 studies were included in this meta-analysis.^15–29^

### Study Characteristics

Characteristics of the studies, including sample size, operative technique, and outcomes, are provided in Table 1. All 15 studies that reported clinical outcomes were observational studies. Eight studies were from the United States^15,16,20,22,23,25–27^; four studies from Italy^17,24,28,29^; one study from Korea^18^; one study from United Kingdom^19^; and one study from Germany.^21^ The sample size ranged from 63 to 642 patients. Fifteen studies included 3101 patients, where 1642 (52.9%) underwent subpectoral IBBR. The follow-up time ranged from 12 to 60 months. The mean follow-up interval was 19.12 months. The mean BMI was significantly higher in the prepectoral IBBR compared to the subpectoral IBBR (25.6 vs. 23.4; *P* < 0.01; Table 1).

**Table 1:** Characteristics of included studies for analysis of prepectoral IBBR versus subpectoral IBBR

Author, yr, Country	Study type	Patients			BMI (kg/m^2)^		Mean age		Outcomes	Mean follow-up (mon)
		Cohort	SR	PP	SR	PP	SR	PP		
Walker et al.^15^ 2021, USA	R	195	103	92	27.8	30.2	55.5	53.0	Complications rate, quality of life	13
Manrique et al.^16^ 2018, USA	R	169	69	100	26.3	25.3	34.2	35.3	Complications rate	17.7
Ribuffo et al.^17^ 2020, Italy	R	642	509	207	24.6	25.3	55.7	56.2	Complications rate	22.1
Yang et al.^18^ 2019, Korea	R	79	47	32	21.2	23.5	46.4	48.9	Complications rate	12
Chandarana et al.^19^ 2018, UK	R	130	69	61	25.1	27.3	50	51	Complications rate	12
Manrique et al.^20^ 2019, USA	R	85	42	33	24.9	25.8	47	54	Complications rate, quality of life	20.6
Thangarajah et al.^21^ 2019, Germany	R	63	29	34	24.4	24.7	49.3	49.9	Complications rate, quality of life	18
King et al.^22^ 2021, USA	R	405	202	203	23.7	24.0	45.9	46.5	Complications rate	24
Plachinski et al.^23^ 2021, USA	R	186	103	83	28.1	26.1	49.9	47.8	Complications rate	18.5
Franceschini et al.^24^ 2021, Italy	R	177	95	82	24.7	23.9	44	47	Complications rate, quality of life, recurrence rate	18
Sinnott et al.^25^ 2018, USA	R	374	100	274	25.2	29.0	46.9	52.4	Complications rate, recurrence rate	25.5
Nealon et al.^26^ 2020, USA	R	256	142	114	25.6	27.4	50.7	52.7	Complications rate, recurrence rate	24.4
Mirhaidari et al.^27^ 2019, USA	R	129	67	62	26.4	27.2	48	54	Complications rate, recurrence rate	24
Cattelani et al.^28^ 2017, Italy	P	86	45	39	26.1	24.9	52.3	52.9	Complications rate, quality of life	12
Bernini et al.^29^ 2015, Italy	P	63	29	34	23	23	51	47	Complications rate, quality of life, recurrence rate	25

*SR *subpectoral IBBR;* PP *prepectoral IBBR;* R *retrospective comparative study;* P* prospective comparative study

### Risk-of-Bias and Publication Bias Assessment

Publication bias was not detected for any of the outcomes investigated in the meta-analysis comparing prepectoral and subpectoral IBBR (eTable 1 in the *Supplement*). Publication bias analysis was not performed for animation deformity and recurrence rates due to shortage of study numbers. Bias in the selection of participants, bias in measurement classification of interventions, bias due to deviation from intended interventions, and bias in selection of the reported result were generally low (eTable 2 in the *Supplement*).

### Prepectoral IBBR versus Subpectoral IBBR: Meta-analysis

#### Overall Complication

**Fig. 2:**
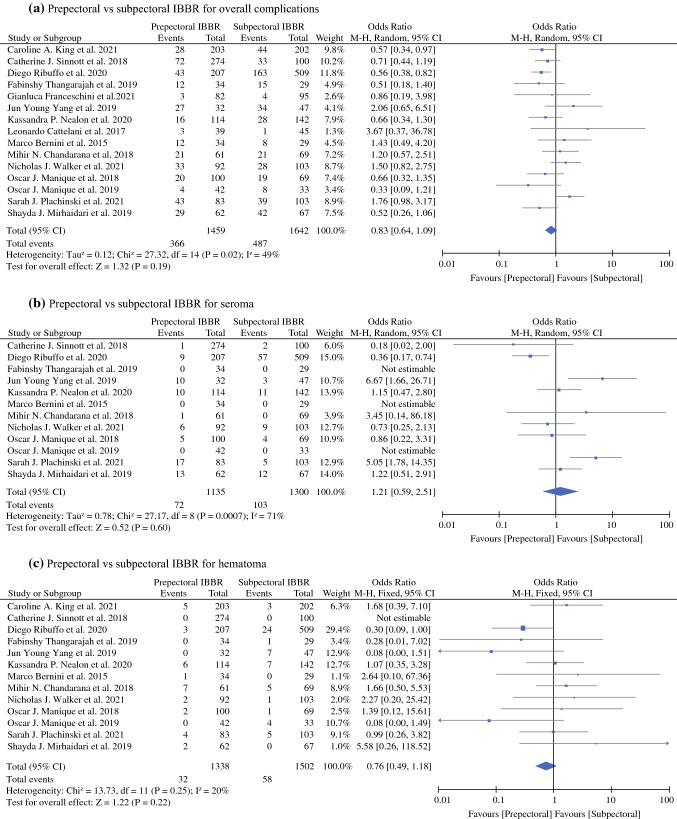
Forest plot comparisons

All 15 studies reporting the overall complications were included in the meta-analysis.^15–29^ The overall complication rates for breasts undergoing prepectoral IBBR was 25.08% (366/1459) and subpectoral IBBR was 29.65% (487/1642). As shown in (Fig. 2a), no significant difference in overall complication rates between prepectoral and subpectoral IBBR was found, with pooled (OR, 0.83; 95% CI, 0.64–1.09; *P* = 0.19). The pooled analysis was performed using a random-effects model, because moderate heterogeneity (*P* = 0.02, *I*^2^ = 49%) among the studies was found.

#### Seroma

Twelve studies in the meta-analysis reported seroma rates.^15–21,23,25–27,29^ As shown in Fig. 2b, no significant difference in seroma rates between prepectoral and subpectoral IBBR was found, with pooled (OR, 1.21; 95% CI, 0.59–2.51; *P* = 0.60). The analysis was performed using a random-effect model, as substantial heterogeneity (*P* = 0.0007, *I*^2^ = 71%) among the studies was found.

#### Hematoma

Thirteen studies reporting data for hematoma rates were included in the meta-analysis.^15–23,25–27,29^ As shown in (Fig. 2c), no significant difference in hematoma rates between prepectoral and subpectoral IBBR was found, with pooled (OR, 0.76; 95% CI, 0.49–1.18; *P* = 0.22). The analysis was performed using a fixed-effect model, as minimal heterogeneity (*P* = 0.25, *I*^2^ = 20%) among the studies was found.

#### Capsular Contracture

**Fig. 3:**
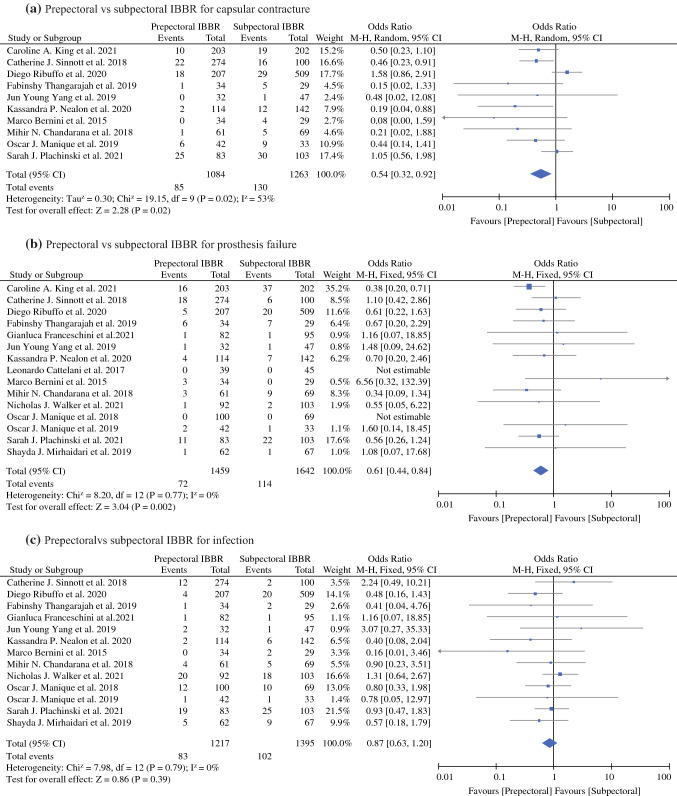
Forest plot comparisons

Ten studies in the meta-analysis reported the capsular contracture rates.^17–23,25,26,29^ As shown in Fig. 3a, our pooled analysis showed that subpectoral IBBR had significantly higher rates of capsular contracture compared to prepectoral IBBR, with pooled (OR, 0.54; 95% CI, 0.32–0.92; *P* = 0.02). The analysis was performed using a random-effect model, as substantial heterogeneity (*P* = 0.02, *I*^2^ = 53%) among the studies was found.

#### Prosthesis Failure

All 15 studies reporting prosthesis failure were included in the meta-analysis.^15–29^ As shown in Fig. 3b, our pooled analysis showed that subpectoral IBBR had significantly higher rates of prosthesis failure compared with prepectoral IBBR, with pooled (OR, 0.61; 95% CI, 0.44–0.84; *P* = 0.002). The pooled analysis was performed using a fixed-effects model, because no significant heterogeneity among the studies was found (*P* = 0.77, *I*^2^ = 0%).

#### Infection

Thirteen studies reporting data for infection rates were included in the meta-analysis.^15–21,23–27,29^ As shown in Fig. 3c, no significant difference in infection rates between prepectoral and subpectoral IBBR was found, with pooled (OR, 0.87; 95% CI, 0.63–1.20; *P* = 0.39). The pooled analysis was performed using a fixed-effects model, because no significant heterogeneity among the studies was found (*P* = 0.79, *I*^2^ = 0%).

#### Skin Flap Necrosis

**Fig. 4:**
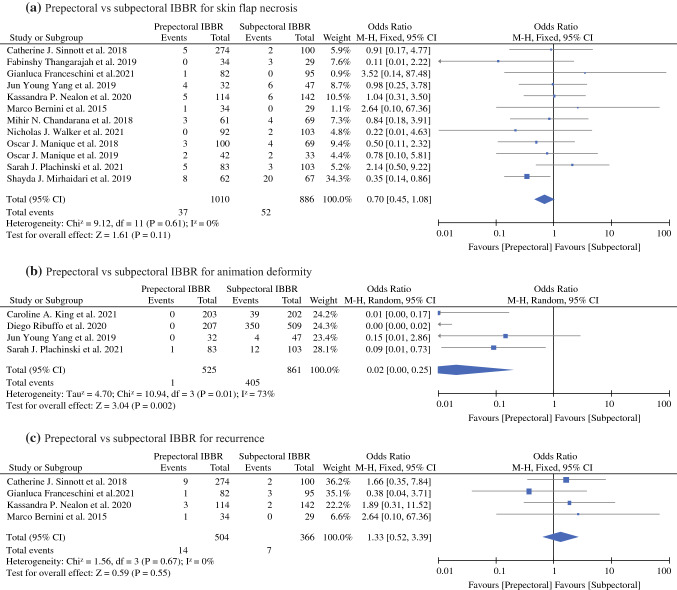
Forest plot comparisons

Twelve studies reporting data for skin flap necrosis were included in the meta-analysis.^15,16,18–21,23–27,29^ As shown in Fig. 4a, no significant difference in skin flap necrosis rates between prepectoral and subpectoral IBBR was found, with pooled (OR, 0.70; 95% CI, 0.45–1.08; *P* = 0.11). The pooled analysis was performed using a fixed-effects model, because no significant heterogeneity among the studies was found (*P* = 0.61, *I*^2^ = 0%).

#### Animation Deformity

Four studies reporting animation deformity were included in the meta-analysis.^17,18,22,23^ As shown in Fig. 4b, our pooled analysis showed that subpectoral IBBR had significantly higher rate of animation deformity compared to prepectoral IBBR, with pooled (OR, 0.02; 95% CI, 0.00–0.25; *P* = 0.002). The analysis was performed using a random-effect model, as substantial heterogeneity (*P* = 0.01, *I*^2^ = 73%) among the studies was found.

#### Oncological Safety

Four studies reporting recurrence were included in the meta-analysis.^24–26,29^ The recurrence rates for breasts undergoing prepectoral IBBR were 2.77% (14/504) and subpectoral IBBR was 1.91% (7/366). As shown in Fig. 4c, no significant difference in recurrence rates between prepectoral and subpectoral IBBR was found, with pooled (OR, 1.31; 95% CI, 0.52–3.39; *P* = 0.55). The pooled analysis was performed using a fixed-effects model, because no significant heterogeneity among the studies was found (*P* = 0.67, *I*^2^ = 0%). However, there were large diffrences in the mean follow-up time between the two groups (prepectoral, 20.4 [range 16–25] months; subpectoral, 27.6 [range 20–35.4] months).^16,19,21,23^

#### Quality of Life

Six studies reporting patient’s quality of life were included in the meta-analysis.^15,20,21,24,28,29^ Two of these studies used postoperative quality of life measuring (QoL): (1) aesthetic satisfaction; (2) skin sensibility; (3) compromised relationship life; (4) sports before surgery; (5) sports after surgery; (6) chronic pain in the pectoral region, and (7) impaired arm motility.^15,24^ Franceschini et al.^24^ reported significant difference in aesthetic satisfaction (*P* < 0.001), skin sensibility (*P* = 0.025) and chronic pain in the pectoral region (*P* < 0.001) in favor of prepectoral IBBR. Four of these studies assessed quality of life using the BREAST-Q, a module measuring post reconstruction satisfaction on five subscales: (1) sexual well-being; (2) satisfaction with the breast; (3) psychosocial well-being; (4) physical well-being; and (5) satisfaction with the outcome.^20,21,28,29^ For each scale, the items responses were summed and transformed into a score, ranging from 0 to 100. Of the four studies that reported comparative BREAST-Q data, only two measured the five subscales. Four of the included studies presented data regarding “satisfaction with breast” subscale. Overall, the scores on “satisfaction with breast” were good for both reconstruction techniques with 77.3% in the prepectoral IBBR group and 71.1% in the subpectoral IBBR group. As shown in eFig. 1A in the Supplement, no significant difference in “satisfaction with breasts” subscale between prepectoral and subpectoral IBBR was found, with pooled (mean difference [MD], 6.55; 95% CI, −1.94–15.04; *P* = 0.13). The pooled analysis was performed using a random-effects model, because considerable heterogeneity among the studies was found (*P* = 0.0002, *I*^2^ = 85%). Similarly, no significant difference was found in the subscales: satisfaction with outcome (eFig. 1B in the Supplement), sexual well-being (eFig. 1C in the Supplement), psychosocial well-being (eFig. 1D in the Supplement), and physical well-being (eFig. 1E in the Supplement).

## Discussion

Breast reconstruction rates have increased over the last decade in the world due to breast cancer and breast cancer prophylaxis.^4^ Prepectoral implant-based breast reconstruction (IBBR) has emerged as an alternative to subpectoral IBBR for the surgical treatment of breast cancer, as it has resurged in popularity, with a growing prominence on the achievement of excellent aesthetics results and improved quality of life without compromising oncologic safety. This meta-analysis provides comparisons with minimum of 12 months follow-up period reporting patient outcomes results following prepectoral IBBR and subpectoral IBBR.

A previous meta-analysis compared prepectoral with subpectoral IBBR based on a pooled analysis of 1,838 patients from 16 comparative studies.^30^ Similar to our results, the authors found that subpectoral IBBR had higher rates of capsular contracture compared with prepectoral IBBR. Moreover, they found no differences in overall complications, seroma, and hematoma rates. However, they suggested that prepectoral IBBR was associated with better Breast-Q scores and a lower rate of skin flap necrosis, which was not observed in our larger analysis.

One of the main goals of implant-based breast reconstruction is to improve the quality of life of patients. Well-developed measurement tools such as the Breast-Q have made it possible to directly compare different breast reconstruction types. Le et al.^31^ showed that quality of life, which was reported using the BREAST-Q subscales (satisfaction with breast, satisfaction with outcome, psychosocial well-being, sexual well-being, and physical well-being) had comparable BREAST-Q satisfaction scores for most modules regardless of implant plane between prepectoral and subpectoral IBBR. This is similar to what we observed after data extraction from the included studies (eFig. 1 in the Supplement).

Our results regarding the association between capsular contracture and lower odds for prepectoral IBBR compared to subpectoral IBBR are consistent with previous research.^30^ In women with locally advanced breast cancer, adjuvant radiotherapy was shown to decrease local recurrence and improve survival in patients with node-positive disease. Despite its therapeutic advantages, adjuvant radiotherapy represents serious risk factors for major complications, such as capsular contracture and reconstructive failure in IBBR. Our findings also have shown that adjuvant radiotherapy is associated with a worse cosmetic satisfaction and higher rate of prosthesis failure.^15,20,21,24,28,29^ It is important to note that the longer duration of follow-up can increase the rate of capsular contracture, as the degree of capsular contracture often increases after the first 3 years.^21,25,28,29^ In patients with IBBR, the contracture affect the skin, capsule, and muscle. It has been suggested that fibrosis of contractile muscle tissue could predispose patients after subpectoral reconstruction to breast contracture and implant deformation.^32^ Sinott et al.^25^ revealed that adjuvant radiotherapy increases the rate of capsular contracture in both groups: subpectoral IBBR (from 2.9 to 52.2%) and prepectoral IBBR (from 3.5 to 16.1%). Sobti et al.^7^ showed that prepectoral IBBR is associated with a lower rate of capsular contracture in an irradiated patient population compared with subpectoral breast reconstruction.

We found that subpectoral IBBR was associated with a higher rate of animation deformity. Fracol et al.^33^ showed that animation deformity is estimated to occur anywhere from 75 to 100% of the subpectoral IBBR. Animation deformity in subpectoral IBBR is caused by contraction of the pectoralis muscle against the breast implant, causing it and the overlying breast shape to shift unnaturally with muscle contraction. This common adverse effect has a large impact on aesthetics, quality of life, and functional comfort. Becker et al.^34^ revealed that approximately half of women with animation deformity experienced disruption to simple activities of daily living. Up to 28% of breast reconstruction patients will request revisionary surgery due to animation deformity, and half of the patients stated that they would have liked to know about alternative surgical options to avoid animation deformity at the time of mastectomy.^33–36^ We also found that prepectoral IBBR was associated with a decrease in prosthesis failure compared with subpectoral IBBR. Accordingly, it is plausible that prepectoral IBBR may be associated with improved long-term outcomes.

Based on our results, there was no difference in skin flap necrosis rates between prepectoral and subpectoral IBBR. In patients with implant-based breast reconstruction, there are several associated risk factors that increase skin flap necrosis such as: smoking, age, hypertension, previous scars, diabetes, radiotherapy, obesity, increased breast volume, and severe comorbidities.^37,38^ Endara et al.^39^ and Daar et al.^40^ showed that the surgical technique also is very important factor for decrease of skin flap necrosis. The main surgical factors that increase skin necrosis are: incision type and decreased mastectomy skin-flap thickness. Daar et al.^40^ also performed a systematic literature review and meta-analysis, including 51 studies with 9,975 NSM, and identified that inframammary incision (IMF) could be the preferred choice with fewer complication and better aesthetic outcomes with a nipple-areola complex (NAC) necrosis rate of 4.62%. In a study by King et al.,^22^ surgeons performed inframammary incision (IMF) in 93.3% (378/405) of cases, with a skin-flap necrosis rate of 4.04%. Several studies have demonstrated the potential benefits of implant-based breast reconstruction and oncological outcomes. Fujihara et al.^41^ showed that the local recurrence rate after implant-based breast reconstruction was 3.1%. In our analysis, the recurrence rates for breasts undergoing prepectoral IBBR were 2.77% (14/504) and subpectoral IBBR was 1.91% (7/366). However, the lack of included studies reporting long-term data hindered our ability to properly assess the long-term oncologic outcomes for prepectoral IBBR versus the subpectoral IBBR.

### Limitations

This study has limitations. The most serious of which was the variation in the sample size among the included studies. Although we analyzed 3101, the sample size ranged widely among the studies from 63 to 642 patients. The included studies were observational in design with limited data on long-term oncological outcomes. The impact of adjuvant therapy on surgical outcomes following implant-based breast reconstruction was conducted by limited number of studies and introduces a risk for bias. Several other factors that were not considered also could affect the outcomes, including different follow-up durations between prepectoral and subpectoral IBBR. This analysis did not give specific data on the implant visibility and rippling between two groups due to lack of included studies reporting data. Future research should include randomized, clinical trials or well-designed, prospective, matched studies with adequate follow-up to assess long-term outcomes between comparative groups. This will help us to choose the most suitable method between the prepectoral and subpectoral IBBR.

## Conclusions

The results of our systematic review and meta-analysis demonstrated that prepectoral, implant-based, breast reconstruction is a safe modality and has similar outcomes with significantly lower rates of capsular contracture, prosthesis failure, and animation deformity compared with subpectoral, implant-based, breast reconstruction.

## Supplementary Information

Below is the link to the electronic supplementary material.

Supplementary file1 (DOC 157 kb)
