# Supplementary material for: Prepectoral Versus Subpectoral Implant-Based Breast Reconstruction: A Systemic Review and Meta-analysis
Source: Ann Surg Oncol. 2022 Oct 16;30(1):126–36. doi: 10.1245/s10434-022-12567-0 (PMC9726796; doi:10.1245/s10434-022-12567-0)
Supplement: Supplementary file 1 — Supplementary file1 (DOC 157 kb) [file 10434_2022_12567_MOESM1_ESM.doc]

**Supplemental Online Content**

Ostapenko E, Nixdorf L, Devyatko Y, et al. Prepectoral versus subpectoral implant-based breast reconstruction: A systemic review and meta-analysis.

**eMethods.** Search Strategy

**eTable 1**. Publication Bias

**eTable 2**. Risk of Bias Assessment

**eFigure 1**. Forest Plot of BREAST-Q

**eReferences.**

The supplemental material has been provided by the authors to give readers additional information about their work.

**Search Strategy**

**DATABASE SEARCHED & TIME PERIOD COVERED: PUBMED: 2011-2021**

**421 results**

**Search Strategy**

(Randomized) OR ("control")) OR (trial)) OR (comparative)) OR (prospective))AND

(Breast neoplasms [MESH terms]) OR ("Breast cancer")) OR ("Breast cancer gene 1")) OR ("Breast cancer gene 2")) OR ("BRCA1")) OR ("BRCA2")) OR (Breast cancer mutation)))

AND

("Prepectoral reconstruction") OR (Prepectoral implant-based breast reconstruction)) OR (Prepectoral IBBR)) OR (Prepectoral immediate breast reconstruction)) OR (Prepectoral immediate IBBR*)) OR (Subpectoral reconstruction*)) OR (Subpectoral implant-based breast reconstruction)) OR (Subpectoral IBBR)) OR (Subpectoral immediate breast reconstruction)) OR (Retropectoral reconstruction)) OR (Retropectoral implant-based breast reconstruction)) OR (Retropectoral IBBR)) OR (Retropectoral immediate breast reconstruction)) OR (implant-based breast reconstruction)) OR (“IBBR“)) OR (Breast reconstruction)) OR (Immediate implant-based breast reconstruction)) OR (Direct to implant breast reconstruction)) OR (single-stage breast reconstruction)) OR (Direct to implant prepectoral breast reconstruction)) OR (Direct to implant subpectoral breast reconstruction)) OR (mastectomy)) OR (nipple-sparing mastectomy)) OR (skin sparing mastectomy)) OR (”NSM”)) OR (“SSM“)) OR (“NAC“)) OR (Nipple-areola complex)) OR (Prophylactic)) OR (Prophylactic mastectomy)) OR (Prophylactic NSM)) OR (Prophylactic SSM)) OR (Ptotic breast)) OR (Large breast)) OR (Breast-Q)) OR (Breast postoperative questionnaire)))Filters: from 2011 – 2021

**DATABASE SEARCHED & TIME PERIOD COVERED:**

**COCHRANE Reviews: 2011 – 2021**

**19 results**

| #1 | (Randomized OR control OR prospective OR comparative OR trial) |
| --- | --- |
| #2 | (Breast neoplasms OR Breast cancer OR Breast cancer gene 1 OR Breast cancer gene 2 OR BRCA1 OR BRCA2 OR (Breast cancer mutation) |
| #3 | (Prepectoral reconstruction OR Prepectoral implant-based breast reconstruction OR Prepectoral IBBR OR Prepectoral immediate breast reconstruction OR Prepectoral immediate IBBR OR Subpectoral reconstruction OR Subpectoral implant-based breast reconstruction OR Subpectoral IBBR OR Subpectoral immediate breast reconstruction OR Retropectoral reconstruction OR Retropectoral implant-based breast reconstruction OR Retropectoral IBBR OR Retropectoral immediate breast reconstruction OR implant-based breast reconstruction OR IBB OR Breast reconstruction OR Immediate implant-based breast reconstruction OR Direct to implant breast reconstruction OR single-stage breast reconstruction OR Direct to implant prepectoral breast reconstruction OR Direct to implant subpectoral breast reconstruction OR mastectomy OR nipple-sparing mastectomy OR skin sparing mastectomy OR NSM OR SSM OR NAC OR Nipple-areola complex OR Prophylactic OR Prophylactic mastectomy OR Prophylactic NSM OR Prophylactic SSM OR Ptotic breast OR Large breast OR Breast-Q OR Breast postoperative questionnaire) |
| #4 | English |
| #5 | Between 1 January 2011 and 31 December 2021 |
| #1 AND #2 AND #3 AND #4 AND #5 | |

**eTable 1**. **Publication Bias**

|  | **Egger regression test (p-value)** |
| --- | --- |
| Overall complication | 0.435 |
| Seroma | 0.836 |
| Hematoma | 0.675 |
| Capsular contracture | 0.156 |
| Prosthesis failure | 0.093 |
| Infection | 0.510 |
| Skin flap necrosis | 0.947 |

**eTable 2. Risk of Bias Assessment Based on ROBINS-I Tool**

| **Author, year** | **Confounding** | **Selection bias** | **Bias in measurement classification of interventions** | **Bias due to deviations from intended interventions** | **Bias due to missing data** | **Bias in measurement of outcomes** | **Bias in selection of the reporter results** | **Other source of bias** |
| --- | --- | --- | --- | --- | --- | --- | --- | --- |
| **Nicholas J. Walker et al.**^1^ **2021** | **Low** | **Moderate** | **Low** | **Low** | **Low** | **Low** | **Low** | **n/a** |
| **Oscar J. Manrique et al.**^2^ **2018** | **Moderate** | **Moderate** | **Low** | **Low** | **Low** | **Low** | **Low** | **n/a** |
| **Diego Ribuffo et al.**^3^ **2020** | **Low** | **Moderate** | **Low** | **Low** | **Low** | **Low** | **Moderate** | **n/a** |
| **Jun Young Yang et al.**^4^ **2019** | **Low** | **Serious** | **Low** | **Low** | **Low** | **Low** | **Moderate** | **n/a** |
| **Mihir N. Chandarana et al.**^5^ **2018, UK** | **Low** | **Serious** | **Low** | **Low** | **Low** | **Low** | **Low** | **n/a** |
| **Oscar J Manrique et al.**^6^ **2019** | **Low** | **Moderate** | **Low** | **Low** | **Low** | **Low** | **Low** | **n/a** |
| **Fabinshy Thangarajah et al.**^7^ **2019** | **Low** | **Moderate** | **Low** | **Low** | **Low** | **Low** | **Low** | **n/a** |
| **Caroline A. King et al.**^8^ **2021** | **Serious** | **Serious** | **Low** | **Low** | **Low** | **Moderate** | **Low** | **n/a** |
| **Sarah J. Plachinski et al.**^9^ **2021** | **Low** | **Moderate** | **Low** | **Low** | **Low** | **Low** | **Low** | **n/a** |
| **Gianluca Franceschini et al.**^10^ **2021** | **Serious** | **Moderate** | **Low** | **Low** | **Low** | **Moderate** | **Moderate** | **n/a** |
| **Catherine J. Sinnott et al.**^11^ **2018** | **Low** | **Moderate** | **Low** | **Low** | **Low** | **Low** | **Low** | **n/a** |
| **Kassandra P. Nealon et al.**^12^ **2020** | **Low** | **Moderate** | **Low** | **Low** | **Low** | **Low** | **Low** | **n/a** |
| **Shayda J. Mirhaidari et al.**^13^ **2019** | **Moderate** | **Moderate** | **Low** | **Low** | **Low** | **Low** | **Low** | **n/a** |
| **Leonardo Cattelani et al.**^14^ **2017** | **Moderate** | **Low** | **Low** | **Low** | **Low** | **Moderate** | **Low** | **n/a** |
| **Marco Bernini et al.**^15^ **2015** | **Low** | **Low** | **Low** | **Low** | **Low** | **Low** | **Low** | **n/a** |

**eFigure 1**. **Forest Plot of BREAST-Q**

1. **Satisfaction with breast**

**
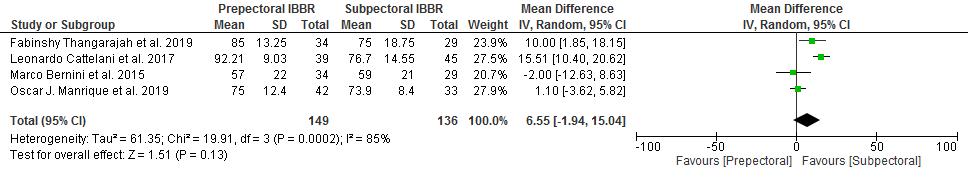
**

1. **Satisfaction with outcome**

**
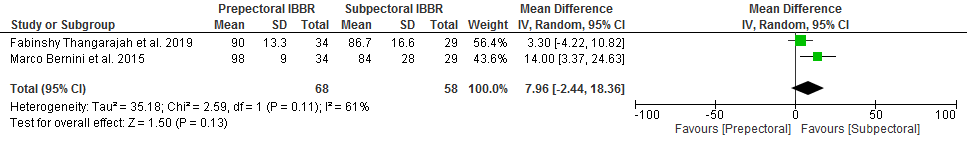
**

1. **Sexual well-being**

**
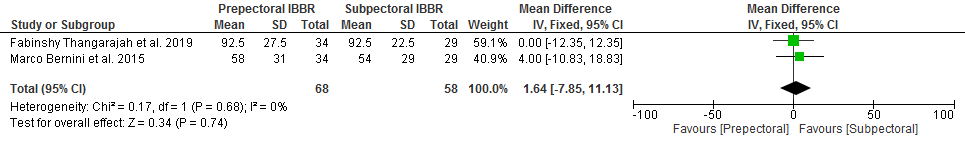
**

1. **Psychosocial well-being**

**
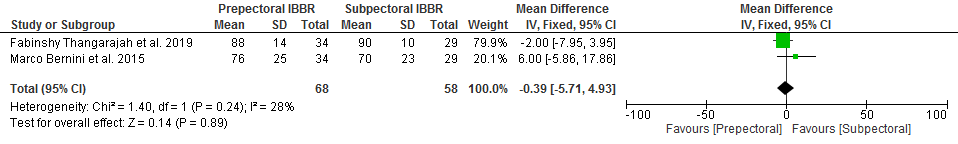
**

1. **Physical well-being**

**
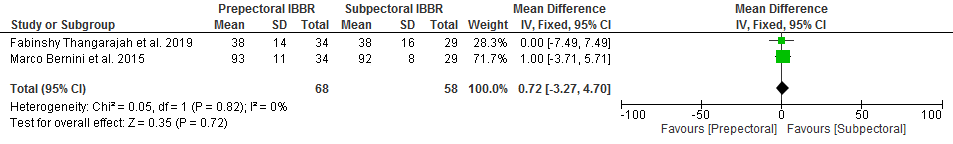
**

**References:**

1. Walker, N. J., Park, J. G., Maus, J. C., *et al.* Prepectoral Versus Subpectoral Breast Reconstruction in High-Body Mass Index Patients. *Ann Plast Surg* 87, 136–143 (2021).

2. Manrique, O. J., Banuelos, J., Abu-Ghname, A., *et al.* Surgical Outcomes of Prepectoral Versus Subpectoral Implant-based Breast Reconstruction in Young Women. *Plast Reconstr Surg Glob Open* 7, e2119 (2019).

3. Ribuffo, D., Berna, G., De Vita, R., *et al.* Dual-Plane Retro-pectoral Versus Pre-pectoral DTI Breast Reconstruction: An Italian Multicenter Experience. *Aesthetic Plast Surg* 45, 51–60 (2021).

4. Yang, J. Y., Kim, C. W., Lee, J. W., *et al.* Considerations for patient selection: Prepectoral versus subpectoral implant-based breast reconstruction. *Arch Plast Surg* 46, 550–557 (2019).

5. Chandarana, M. N., Jafferbhoy, S., Marla, S., Soumian, S. & Narayanan, S. Acellular dermal matrix in implant-based immediate breast reconstructions: a comparison of prepectoral and subpectoral approach. *Gland Surgery* 7, S64-S6S69 (2018).

6. Manrique, O. J., Kapoor, T., Banuelos, J., *et al.* Single-Stage Direct-to-Implant Breast Reconstruction: A Comparison Between Subpectoral Versus Prepectoral Implant Placement. *Ann Plast Surg* 84, 361–365 (2020).

7. Thangarajah, F., Treeter, T., Krug, B., *et al.* Comparison of Subpectoral versus Prepectoral Immediate Implant Reconstruction after Skin- and Nipple-Sparing Mastectomy in Breast Cancer Patients: A Retrospective Hospital-Based Cohort Study. *Breast Care* 14, 382–387 (2019).

8. King, C. A., Bartholomew, A. J., Sosin, M., *et al.* A Critical Appraisal of Late Complications of Prepectoral versus Subpectoral Breast Reconstruction Following Nipple-Sparing Mastectomy. *Ann Surg Oncol* 28, 9150–9158 (2021).

9. Plachinski, S. J., Boehm, L. M., Adamson, K. A., LoGiudice, J. A. & Doren, E. L. Comparative Analysis of Prepectoral versus Subpectoral Implant-based Breast Reconstruction. *Plast Reconstr Surg Glob Open* 9, e3709 (2021).

10. Franceschini, G., Scardina, L., Di Leone, A., *et al.* Immediate Prosthetic Breast Reconstruction after Nipple-Sparing Mastectomy: Traditional Subpectoral Technique versus Direct-to-Implant Prepectoral Reconstruction without Acellular Dermal Matrix. *J Pers Med* 11, 153 (2021).

11. Sinnott, C. J., Persing, S. M., Pronovost, M., *et al.* Impact of Postmastectomy Radiation Therapy in Prepectoral Versus Subpectoral Implant-Based Breast Reconstruction. *Ann Surg Oncol* 25, 2899–2908 (2018).

12. Nealon, K. P., Weitzman, R. E., Sobti, N., *et al.* Prepectoral Direct-to-Implant Breast Reconstruction: Safety Outcome Endpoints and Delineation of Risk Factors. *Plast Reconstr Surg* 145, 898e–908e (2020).

13. Mirhaidari, S. J., Azouz, V. & Wagner, D. S. Prepectoral Versus Subpectoral Direct to Implant Immediate Breast Reconstruction. *Ann Plast Surg* 84, 263–270 (2020).

14. Cattelani, L., Polotto, S., Arcuri, M. F., *et al.* One-Step Prepectoral Breast Reconstruction With Dermal Matrix-Covered Implant Compared to Submuscular Implantation: Functional and Cost Evaluation. *Clin Breast Cancer* 18, e703–e711 (2018).

15. Bernini, M., Calabrese, C., Cecconi, L., *et al.* Subcutaneous Direct-to-Implant Breast Reconstruction: Surgical, Functional, and Aesthetic Results after Long-Term Follow-Up. *Plast Reconstr Surg Glob Open* 3, e574 (2016).
